# Automated Machine Learning Frameworks for Radiomics: Comparative Evaluation Study

**DOI:** 10.2196/91492

**Published:** 2026-06-11

**Authors:** Jose Lozano-Montoya, Emilio Soria-Olivas, Almudena Fuster-Matanzo, Angel Alberich-Bayarri, Ana Jimenez-Pastor

**Affiliations:** 1 Universitat de València Valencia, Valencia Spain; 2 Research & Frontiers in AI Department Quantitative Imaging Biomarkers in Medicine Valencia, Valencia Spain; 3 Intelligent Data Analysis Laboratory Universitat de València Valencia, Valencia Spain

**Keywords:** automated machine learning, radiomics, comparative study, classification, medical imaging

## Abstract

**Background:**

Automated machine learning (AutoML) frameworks can lower technical barriers for predictive and prognostic model development in radiomics by enabling researchers without programming expertise to build models. However, their effectiveness in addressing radiomics-specific challenges remains unclear.

**Objective:**

This study aimed to evaluate the performance, efficiency, and accessibility of general-purpose and radiomics-specific AutoML frameworks on diverse radiomics classification tasks, thereby guiding researchers and highlighting development needs for radiomics.

**Methods:**

A total of 10 public and private radiomics datasets with varied imaging modalities (computed tomography and magnetic resonance imaging), sizes, anatomies, and end points were used. Six general-purpose and 5 radiomics-specific frameworks were tested with predefined parameters using standardized cross-validation. Evaluation metrics included area under the receiver operating characteristic curve, runtime, and qualitative aspects related to software status, accessibility, and interpretability.

**Results:**

Simplatab, a radiomics-specific tool with a no-code interface, achieved the best overall balance between performance and computational efficiency, recording the highest average test area under the receiver operating characteristic curve (mean 78.46%, SD 12.22%) with a moderate runtime (1.1 h). However, its performance was not statistically superior to the most intensive general-purpose solutions. Most radiomics-specific frameworks were excluded from the performance analysis due to obsolescence, extensive programming requirements, or computational inefficiency. Conversely, general-purpose frameworks demonstrated higher accessibility and ease of implementation.

**Conclusions:**

While no single framework demonstrated absolute predictive superiority, Simplatab provides an effective balance of performance, efficiency, and accessibility for radiomics classification problems. However, continued efforts are needed to further mature AutoML solutions in the radiomics domain.

## Introduction

In recent years, automated machine learning (AutoML) has emerged as a powerful approach to reducing technical barriers in machine learning model development. AutoML refers to the automation of the end-to-end machine learning workflow, including data preprocessing, feature selection, model selection, and hyperparameter optimization [1]. By delegating these technically demanding steps to algorithm-driven processes, AutoML allows clinicians and researchers without extensive machine learning experience to use advanced modeling techniques effectively, enabling medical professionals to concentrate on clinical questions and data interpretation instead of technical model building [2]. Modern AutoML frameworks promise improved model performance while promoting consistency and reproducibility by following structured, algorithm-driven processes that reduce individual variations. Similar to the introduction of genomics into clinical practice, medical professionals need transparent and interpretable models to understand radiomics features and apply them across the full spectrum of clinical end points, including classification tasks (eg, tumor grading and diagnosis), regression problems (eg, predicting continuous biomarkers or treatment response), and survival or time-to-event analyses that are essential in oncology research.

**Figure 1:**
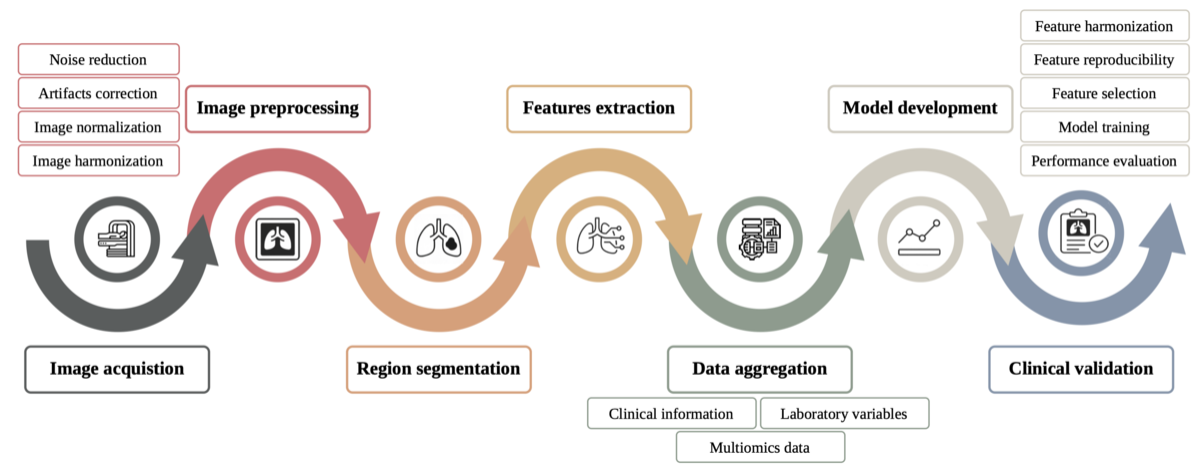
Overview of the standard radiomics pipeline. Image acquisition initiates the radiomics pipeline, followed by preprocessing and segmentation of the region of interest. Quantitative features are extracted, creating high-dimensional datasets. Prior to modeling, features may be harmonized to correct nonbiological variations caused by differences in acquisition conditions, filtered for stability, or reduced. Features can be combined with additional data before training machine learning models to eventually achieve clinical validation.

Radiomics represents a particularly challenging use case for AutoML in medical imaging. It involves the extraction of a large number of quantitative features from imaging exams for tissue characterization to serve as imaging biomarkers correlated with disease diagnosis or clinical end points [3]. The classical radiomics pipeline involves multiple sequential steps requiring domain-specific decisions and multidisciplinary expertise [4] (Figure 1) but faces significant limitations including a lack of reproducibility and standardization, where small variations in image acquisition or processing substantially affect extracted features [5,6]. Critical challenges include the need for harmonization methods to ensure feature consistency across different imaging platforms and institutions [7], and the high dimensionality of radiomics data relative to sample sizes, which creates highly correlated, sparse feature sets prone to overfitting that reduces feature robustness and biological interpretability across studies [8]. Numerous studies have shown the potential of radiomics features and radiomics-based models for cancer detection, tumor grading, and even predicting survival or treatment response, ultimately enhancing clinical decision-making [9-11]. Despite its potential, scaling radiomics into routine clinical practice remains challenging, and to date, no diagnostic tests or companion diagnostics based on radiomics have been successfully implemented.

To address these challenges, several radiomics-specific AutoML frameworks have been developed to explicitly address the complexity of radiomics workflows, automating steps from feature extraction to model building [12,13]. In parallel, general-purpose AutoML frameworks, designed primarily for automating machine learning tasks on tabular data, have also been increasingly applied to radiomics studies. However, these efforts have largely remained method-specific, as most published studies have evaluated a single approach or a limited subset of tools in isolation [12-14]. Likewise, applications of general AutoML libraries to radiomics are often bespoke and tailored to specific use cases, without a unified methodological perspective across paradigms. [11]. As a result, current evidence provides only a fragmented view of how AutoML strategies behave in radiomics, particularly with respect to robustness, performance stability, and computational trade-offs across heterogeneous scenarios.

In this work, we explore the behavior of different AutoML frameworks in radiomics-based medical image classification. Using a unified experimental setup across 10 heterogeneous radiomics datasets, we compare the performance and computational characteristics of both general-purpose and radiomics-oriented AutoML frameworks across diverse imaging modalities, anatomical regions, and clinical end points. Our ultimate goal is to provide methodological insights that help guide the design and practical deployment of AutoML solutions in radiomics, supporting more robust, reproducible, and clinically relevant modeling workflows.

## Methods

### Ethical Considerations

This retrospective computational evaluation was conducted in accordance with the ethical principles of the Declaration of Helsinki. For the 8 publicly available datasets used, original study approvals apply [15,16]. For the private “Prostate” dataset, data were obtained from the ProCAncer-I project (grant 952159) [17]. Access was granted under the ProCAncer-I Data Sharing Agreement, which ensured full anonymization of the data and waived the requirement for informed consent for retrospective data use. For the private “Lung” dataset, given the use of retrospective and deidentified data, the study did not require institutional review board approval.

### Datasets

This study used 10 distinct radiomics datasets to evaluate the behavior of AutoML frameworks in radiomics-based classification tasks. These datasets included diverse imaging modalities, anatomical regions, varying class balances, and clinical end points, thereby offering comprehensive coverage of the radiomics applications (Table 1 and Table S1 in Multimedia Appendix 1 [18,19]). Eight publicly available datasets from Open Radiomics (Brain Tumor Segmentation [BraTS] and The Cancer Imaging Archive [TCIA]) [15] and Workflow for Optimal Radiomics Classification (WORC; colorectal liver metastases [CRLM], Desmoid, gastrointestinal stromal tumors [GIST], Lipo, Liver, and Melanoma) databases were included [16]. Additionally, 2 private institutional datasets were added to test the generalization in radiomics contexts not represented in the public domain (Prostate [17] and Lung). Patient cohorts ranged significantly in size from 74 to 577 participants, reflecting variability between smaller single-institution datasets and larger multicenter collections. The inclusion and exclusion criteria for each cohort are detailed in their respective original publications [15-17]. To ensure standardized reporting and methodological transparency for the AI components of this study, we have provided the CLAIM (Checklist for Artificial Intelligence in Medical Imaging) reporting checklist [18] as Table S2 in the Multimedia Appendix 1.

All datasets provided preextracted radiomics features compliant with the Image Biomarker Standardization Initiative. Therefore, the present evaluation focuses exclusively on the modeling and classification stages of the radiomics workflow, and not on the full end-to-end pipeline including image preprocessing, segmentation, and feature extraction.

**Table 1:** Summary of radiomics datasets included in the comparative analysis.

Dataset (accessibility)		Size, n	Image modality	Region of interest	Radiomic feature segmentation	Radiomic features extracted, n	Clinical variables, n	Binary target
**Open Radiomics (public)**								
	BraTS^a^	577	MR^b^-T1w	Brain: primary tumor	Manual	1688	0^c^	MGMT^d^: methylated or nonmethylated
	TCIA^e^	421	CT^f^	NSCLC^g^: primary tumor	Manual	1688	3 (2 categorical)	TNM^h^ overall stage: I and II or III and IV
**WORC** ^i^ **(public)**								
	CRLM^j^	74	CT	Colorectal: metastatic	Semiautomatic	1379	2 (1 categorical)	HGP^k^: desmoplastic or replacement
	Desmoid	202	MR-T1w	Soft tissue: primary tumor	Semiautomatic	1379	2 (1 categorical)	DTF^l^ or STS^m^
	GIST^n^	246	CT	Gastrointestinal: lesion	Semiautomatic	1379	2 (1 categorical)	GIST or no-GIST
	Lipo	114	MR-T1w	Fat tissue: lesion	Semiautomatic	1379	2 (1 categorical)	Lipoma or WDLPS^o^
	Liver	185	MR-T2w	Liver: primary tumor	Semiautomatic	1379	2 (1 categorical)	Malignant or benign
	Melanoma	102	CT	Lung: metastatic nodules	Semiautomatic	1379	2 (1 categorical)	BRAF^p^: mutated or wild type
Lung (private)		554	CT	NSCLC: primary tumor and lymph nodes	Semiautomatic	1379	36 (2 categorical)	Survival at 12 months
Prostate (private)		333	MR-T2w	Prostate: central and transition zones	Semiautomatic	1379	0	Gleason < 7 or gleason 7

^a^BraTS: Brain Tumor Segmentation.

^b^MR: magnetic resonance.

^c^Zero occurrences.

^d^MGMT: O^6^-methylguanine-DNA methyltransferase.

^e^TCIA: The Cancer Imaging Archive.

^f^CT: computed tomography.

^g^NSCLC: non-small cell lung cancer.

^h^TNM: tumor-node-metastasis.

^i^WORC: Workflow for Optimal Radiomics Classification.

^j^CRLM: colorectal liver metastases.

^k^HGP: histopathological growth pattern.

^l^DTF: desmoid-type fibromatosis.

^m^STS: soft-tissue sarcoma.

^n^GIST: gastrointestinal stromal tumors.

^o^WDLPS: well-differentiated liposarcoma.

^p^BRAF: v-raf murine sarcoma viral oncogene homolog B1 gene.

### AutoML Frameworks

AutoML frameworks were selected based on two primary criteria: (1) open-source availability with no licensing fees and (2) prominence in current literature, defined here as recurrent use or discussion in recent peer-reviewed studies within the fields of medical imaging, radiomics, and AutoML. This selection aimed to represent both major AutoML paradigms, namely general-purpose frameworks originally developed for tabular data and domain-specific approaches tailored to radiomics. General-purpose AutoML methods included Autogluon (AWS AI, Amazon Web Services) [20], H2O AutoML (H2O.ai) [21], LightAutoML (Sber AI Lab) [22], MLjar (MLJAR) [23], PyCaret [24], and TPOT (EpistasisLab) [25], while radiomics-specific tools included AutoRadiomics [13], AutoML for Radiomics [26], AutoPrognosis (The van der Schaar Lab) [27], Simplatab [14], and WORC [12].

### Evaluation Criteria

#### Frameworks Assessment

First, 3 qualitative aspects were assessed: obsolescence, accessibility, and explainability. Obsolescence was examined through repository activity and update frequency. Repository status was categorized as “active” (contributions within the last 6 months), “maintenance” (activity between 6 months and 2 years), or “obsolete” (inactive for more than 2 years), while update frequency was specifically rated as “high” (monthly releases), “moderate” (at least every 6 months), or “low” (sporadic updates less than once a year). Accessibility determined the barrier to entry based on installation complexity and required user expertise. Deployment quality was rated “high” for standard Python (Python Software Foundation) packages (accessible via pip, uv, or Docker) with comprehensive documentation, scaling down to “low” for complex builds with sparse guidance. Concurrently, the required learning curve was categorized from “low” for libraries adhering to standard conventions like the scikit-learn application programming interface, to “advanced,” which required deep expertise in the framework. Finally, interpretability was evaluated for the frameworks’ ability to provide model-level explanations of predictions, distinguishing between “advanced” integration of model-agnostic methods (eg, Shapley additive explanations and local interpretable model-agnostic explanations), “basic feature importance reporting,” and “none.” These evaluations were conducted by a data scientist with 4 years of experience developing radiomics-based predictive and prognostic algorithms. Frameworks deemed obsolete or exhibiting low accessibility were excluded from subsequent quantitative analysis.

**Figure 2:**
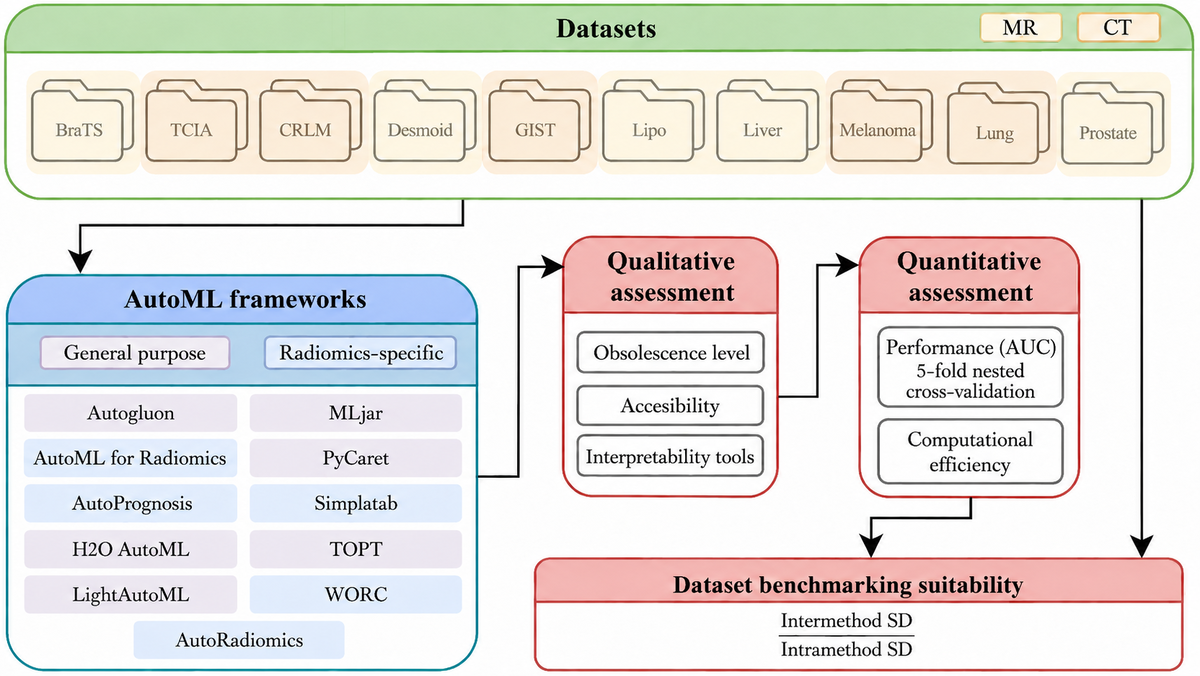
Methodological workflow for the comparative evaluation of automated machine learning (AutoML) frameworks. Frameworks were evaluated through qualitative and quantitative assessment, while also evaluating dataset suitability for benchmarking. Obsolete or inaccessible frameworks were excluded from performance comparison. AUC: area under the receiver operating characteristic curve; BraTS: brain tumor segmentation; CRLM: colorectal liver metastases; CT: computed tomography; GIST: gastrointestinal stromal tumors; MR: magnetic resonance; TCIA: The Cancer Imaging Archive; TPOT: Tree-Based Pipeline Optimization Tool; WORC: Workflow for Optimal Radiomics Classification.

Then, frameworks were quantitatively assessed based on their predictive performance using the area under the receiver operating characteristic curve (AUC) and computational efficiency based on the execution times during experiments. These 2 metrics were jointly analyzed to characterize the trade-off between predictive performance and computational efficiency across frameworks. An efficiency baseline was subsequently defined based on the joint assessment of predictive performance and computational runtime. Autogluon and MLjar offered different predefined performance configurations (presets) that were also evaluated as independent frameworks. Figure 2 summarizes the complete evaluation workflow.

To ensure methodological consistency, all datasets underwent minimal preprocessing to allow each AutoML method to apply its specific training pipeline. Exceptions to this procedure occurred only when certain frameworks had inherent limitations: median imputation for Simplatab (missing value incompatibility) and numerical encoding for the Tree-Based Pipeline Optimization Tool (TPOT; categorical variable incompatibility). These exceptions were implemented to avoid excluding patients or variables, which would have altered the comparison across frameworks. All methods were initialized with predefined parameters and evaluated under the same hardware conditions with identical partitions. Performance was assessed using 5-fold nested cross-validation, reported as mean test AUC (SD) across the 5 folds. In each outer fold, the training partition was used for internal model development and optimization, whereas the held-out test partition was used for final performance estimation.

Additional details regarding radiomics feature extraction, framework pipelines, experimental setup and complementary metrics are provided in Table S3a and Table S3b in the Multimedia Appendix 1. All the code is available in GitHub [28].

#### Dataset Suitability for AutoML Evaluation in Radiomics

An effective dataset for comparative methodological evaluation should enable the measurement of consistent and discriminative signals of methodological differences. We adapted criteria from medical image segmentation validation to assess dataset suitability for AutoML in radiomics [29]. Two main requirements were defined to categorize a dataset as suitable for benchmarking: (1) low SD of AUC scores from the same method across the 5 folds (intramethod SD), which indicates statistical stability by ensuring that a method’s observed performance is consistent and not due to random chance or specific data splits; and (2) a high SD across different methods (intermethod SD), which indicates the presence of meaningful signals of methodological differences. Low intermethod SD would suggest that the task is too simple or that performance saturates quickly, limiting its utility for distinguishing methodological superiority.

The final suitability score was defined as the ratio between intermethod and intramethod SD. Conceptually rooted in the principles of ANOVA, this metric functions as a dimensionless signal-to-noise indicator of how clearly methodological differences can be detected. In this context, the intermethod SD acts as the “signal,” capturing the variance between the methods; while the intramethod SD represents the statistical “noise,” that is, the variance introduced by data resampling during the nested cross-validation. A ratio exceeding a parity threshold of 1.0 indicates that algorithmic superiority is distinct and distinguishable above the inherent noise. Datasets with low intramethod SD and high intermethod SD are preferred as they offer high differentiation power and result stability. Importantly, this metric should be interpreted as an exploratory heuristic rather than a formally validated measure.

### Statistical Analysis

Statistical evaluation of performance differences was conducted using Python 3.11 (Python Software Foundation) and SciPy v.1.12 (SciPy community). Given the multiclassifier, multidataset design, we applied a nonparametric rank-based approach. The Friedman test assessed whether overall performance rankings differed significantly across the datasets, with global effect size quantified by Kendall *W*. Where significant differences were found (*P*<.05), pairwise comparisons were conducted using the Nemenyi post hoc test.

Overall mean AUC was reported as an unweighted macroaverage across all datasets to assess generalization and methodological robustness across clinically heterogeneous tasks, and median paired AUC differences and their 95% CIs were calculated to quantify performance gaps between frameworks.

## Results

### Dataset Suitability for Comparative Methodological Evaluation

**Figure 3:**
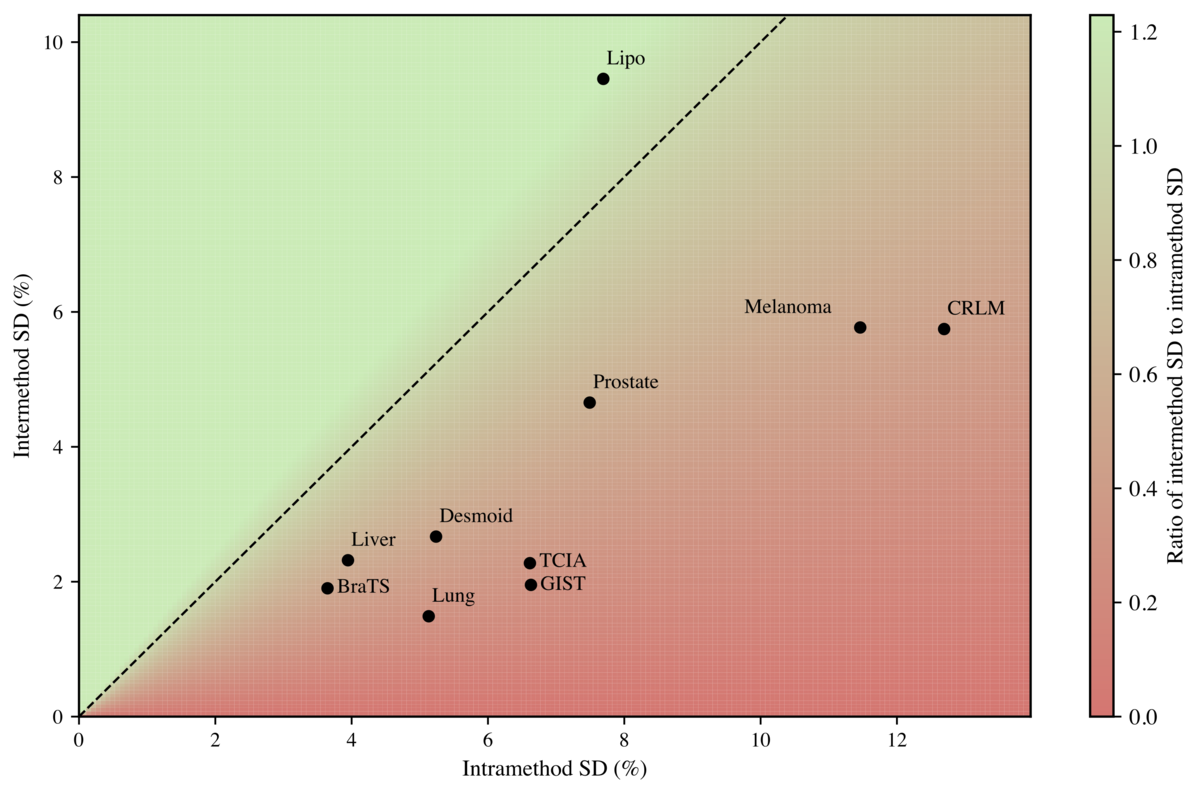
Dataset suitability for comparative methodological evaluation. Suitability was quantified as the ratio between intermethod and intramethod SD based on the mean area under the receiver operating characteristic curve (AUC) obtained across AutoML frameworks. The horizontal axis (intramethod SD%) reflects the internal variability of AUC values within each method across cross-validation folds, whereas the vertical axis (intermethod SD%) represents the variability of AUC values across different methods. The dashed line denotes a ratio of one. Datasets located in the upper-left (green) region combine low internal variability with high intermethod differentiation. BraTS: Brain Tumor Segmentation; CRLM: colorectal liver metastases; GIST: gastrointestinal stromal tumors; TCIA: The Cancer Imaging Archive.

Figure 3 summarizes the overall suitability of the evaluated datasets for comparative methodological analysis, using an exploratory metric defined as the ratio of intermethod to intramethod AUC SD.

As observed, datasets such as Lipo illustrated scenarios with a favorable balance between result stability and intermethod differentiation. Conversely, datasets like CRLM and Melanoma exhibited high intramethod variability, limiting the reliability of performance comparisons. In between these 2 extremes, Prostate exhibited intermediate behavior, with relatively higher internal variability than the most stable datasets. Finally, TCIA, GIST, Desmoid, Lung, Liver, and BraTS demonstrated stable results (low intramethod SD) but limited discriminatory capacity (low intermethod SD) between methods.

### Qualitative Assessment

Table 2 provides a summary of the qualitative characteristics for all initially considered frameworks. The assessment revealed limited viability for several radiomics-specific tools. Therefore, AutoML for Radiomics and AutoRadiomics were classified as obsolete, with inactive repositories and no recent updates, rendering them unusable in the experimental setup. WORC, while actively maintained, proved incompatible with the preextracted radiomics features used in this study and demanded a high level of programming expertise, deviating from the low-code paradigm typical of AutoML. AutoPrognosis, although functional and offering advanced capabilities, exhibited very high computational demands, failing to complete the first cross-validation fold within 72 hours on typical high-dimensional radiomics datasets, which led to its exclusion from the subsequent quantitative analysis. Consequently, only Simplatab remained as a viable radiomics-specific framework for the quantitative comparison.

In contrast, general-purpose frameworks such as LightAutoML, MLjar, and Autogluon demonstrated active maintenance, straightforward installation procedures, and lower requirements for machine learning expertise.

Notably, several frameworks, including MLjar, Simplatab, and TPOT, offered advanced interpretability tools (eg, Shapley Additive Explanations values and model bias analysis), a key differentiator for clinical translation and trustworthiness, whereas others provided only limited or no interpretability functionality.

**Table 2:** Qualitative characteristics of the general-purpose and radiomics-specific automated machine learning frameworks.

Framework	Focus	Type of problem	Obsolescence level		Accessibility			Interpretability tools^a^
			Repository Activity	Update frequency^b^	Ease of installation	Required ML^c^ knowledge		
Autogluon	General purpose	Classification, regression, and time series	Active	High	High	Low	None	
AutoML for Radiomics	Radiomics	Classification	Obsolete	—^d^	Not possible	Intermediate	Basic	
AutoPrognosis	Health care	Classification, regression, and survival	Active	High	High	Low	Advanced	
AutoRadiomics	Radiomics	Classification	Obsolete	Low	Medium	Intermediate	Basic	
H2O AutoML	General purpose	Classification and regression	Active	High	High	Low	None	
LightAutoML	General purpose	Classification and regression	Active	High	High	Low	Basic	
MLjar	General purpose	Classification and regression	Active	High	High	Low	Advanced	
PyCaret	General purpose	Classification, regression, time series, clustering, and anomaly detection	Active	Moderate	High	Low	None	
Simplatab	Radiomics	Classification	Active	Moderate	High	Low	Advanced	
TPOT^e^	General purpose	Classification, regression	Maintenance	Moderate	High	Low	None	
WORC^f^	Radiomics	Classification	Maintenance	Moderate	Not possible	Advanced	None	

^a^Basic: offers basic visualization or basic information about the features of the model, and advanced: includes advanced techniques such as Shapley additive explanations, local interpretable model-agnostic explanations, and heatmap visualization.

^b^High: one or more times per month; moderate: at least every 6 months; and low: less than once a year.

^c^ML: machine learning.

^d^Not applicable.

^e^TPOT: Tree-Based Pipeline Optimization Tool.

^f^WORC: Workflow for Optimal Radiomics Classification.

### Quantitative Performance and Efficiency

The quantitative evaluation was performed on the general-purpose frameworks and Simplatab, following the qualitative filtering described previously. Table 3 presents the detailed AUC results (mean, SD) for each framework across the 10 datasets, alongside the average and the median paired differences in performance compared with the top performer. While AUC was prioritized as a threshold-independent metric, complementary threshold-dependent clinical metrics (sensitivity, specificity, *F*_1_-score, and balanced accuracy) were also computed in Table S4 in Multimedia Appendix 1. Consistent with the AUC findings, the complementary metrics reflected similar performance patterns across frameworks, although absolute values varied depending on the decision thresholds applied by each tool. However, no statistically significant differences were observed. Additionally, the aggregated results excluding CRLM and Melanoma are provided in Table S5 in Multimedia Appendix 1 for comparison, as these datasets were initially excluded due to limited compatibility across several frameworks.

**Table 3:** Results of the comparative evaluation of AutoML frameworks^a^.

	BraTS^b^ (n=577; %), mean (SD)	CRLM^c^ (n=74; %), mean (SD)	Desmoid (n=202; %), mean (SD)	Lipo (n=114; %), mean (SD)	Liver (n=185; %), mean (SD)	Melanoma (n=102; %), mean (SD)	Prostate (n=333; %), mean (SD)	TCIA^d^ (n=421; %), mean (SD)	Lung (n=554; %), mean (SD)	GIST^e^ (n=246; %), mean (SD)	Average AUC (%), (SD)^f^	Median Δ vs Ref (95% CI)^g^	Runtime
Autogluon medium	58.7 (3.5)	63.2 (18)	89.7 (8.7)	85.6 (6.1)	92.5 (4.4)	56.5 (22)	68.8 (9.5)	67.7 (4.6)	79.4 (4.9)	75.3 ( 5.3)	73.72 (12.81)^g^	4.6 (3.0-6.5)	3 min
Autogluon good	60.5 (4.5)	53.1 (17.1)	93.2 (3.9)	81.1 (13.2)	94.9 (2.8)	51.1 (13.2)	67.4 (10.6)	70.0 (7.0)	80.3 (5.9)	78.7 ( 5.9)	73.02 (15.31)	3.8 (2.1-8.7)	1.5 h
Autogluon high	58.9 (5.5)	50.8 (9.2)	92.4 (4.1)	85.5 (9.9)	94.1 (3.4)	50.2 (12.9)	68.1 (9.4)	71.2 (6.8)	80.0 (5.9)	79.7 (5.0)	73.08 ( 16.02)	2.8 (2.4-9.1)	5 h
Autogluon best	57.7 (3.6)	57.3 (8.8)	93.1 (4.0)	80.2 (11.8)	95.0 (3.3)	52.3 (12.9)	70.2 (9.1)	70.2 (7.3)	80.9 (5.2)	79.7 (6.6)	73.66 (14.77)	3.5 (1.9-7.2)	5 h
MLjar explain	58.6 (4.0)	56.3 (11.3)	92.0 (5.4)	83.3 (8.6)	88.6 (5.0)	51.4 (14.3)	63.9 (9.3)	70.0 (8.3)	77.2 (2.9)	75.1 (7.1)	71.64 (13.98)^h^	6.2 (4.4-8.3)	17 min
MLjar perform	59.5 (3.4)	58.4 (11.6)	91.1 (5.5)	81.4 (8.3)	94.2 (2.6)	52.9 (14.6)	68.7 (11.3)	71.6 (7.1)	81.1 (5.1)	78.8 (7.6)	73.78 (13.99)	3.8 (2.5-6.1)	5 h
MLjar compete	59.7 (2.2)	56.0 (20.3)	88.6 (4.0)	76.8 (11.5)	90.0 (5.5)	51.6 (7.6)	70.3 (7.9)	66.9 (6.2)	78.3 (6.1)	78.5 (7.1)	71.67 (13.14)^h^	6.4 (3.8-9.0)	5 h
MLjar optuna	60.5 (3.7)	53.0 (18.1)	90.7 (5.6)	81.7 (8.3)	93.9 (3.3)	43.3 (9.5)	72.5 (4.4)	70.5 (4.5)	80.6 (3.4)	78.4 (6.6)	72.51 (16.19)	3.7 (2.3-7.9)	34 h
H2O AutoML	56.9 ( 4.3)	50.0 (0.0)	85.8 (7.2)	50.0 (0.0)	89.4 (6.3)	50.0 (0.0)	68.2 (5.9)	70.9 (5.3)	78.2 (6.8)	76.5 (8.0)	67.59 (15.10)^g^	6.7 (5.0-14.5)	30 s
LightAutoML	56.7 (4.2)	52.3 (14.2)	92.6 (4.4)	84.4 (3.2)	93.5 (4.6)	41.7 (9.8)	68.9 (3.0)	71.5 (5.3)	80.8 (5.0)	77.2 (7.2)	71.95 (17.29)^h^	3.9 (2.7-8.6)	6 min
PyCaret	56.2 (3.0)	42.8 (12.4)	86.5 (9.6)	81.6 (6.1)	93.0 ( 4.3)	52.2 (15.0)	54.5 (4.7)	65.7 (9.4)	78.8 (5.2)	76.8 (8.6)	68.80 (16.83)^h^	7.8 (4.8-13.7)	16 min
Simplatab	63.3 (2.4)	64.5 (10.0)	95.0 (3.8)	87.7 (5.5)	96.4 (2.3)	65.6 (7.5)	73.3 (5.8)	74.1 (7.1)	82.4 (5.9)	82.3 (4.4)	78.46 (12.22)	—^j^	1.1 h
TPOT^i^	58.4 (3.3)	58.6 (15.0)	91.0 (2.3)	78.2 (8.1)	92.7 (3.7)	49.2 (10.5)	67.3 (7.1)	67.8 (7.9)	81.4 (5.0)	78.2 (7.3)	72.28 (14.46)^h^	5.4 (3.9-7.7)	2.5 h

^a^Performance on individual datasets is reported as mean area under the receiver operating characteristic curve (AUC), % (SD) from 5-fold nested cross-validation.

^b^BraTS: Brain Tumor Segmentation.

^c^CRLM: colorectal liver metastases.

^d^TCIA: The Cancer Imaging Archive.

^e^GIST: gastrointestinal stromal tumors.

^f^Average AUC across datasets (SD).

^g^Median paired differences (Δ vs Ref) in AUC, calculated relative to the top-performing framework (Simplatab), with 95% CIs.

^h^Statistically significant difference in AUC compared with the top performer Simplatab, as determined by the Friedman test (*P*<.001, Kendall *W*=0.568 [strong]) and Nemenyi post hoc test (*P*<.05).

^i^TPOT: Tree-Based Pipeline Optimization Tool.

^j^Not applicable.

Overall, Simplatab achieved the highest average AUC (mean 78.46%, SD 12.22%) across the heterogeneous datasets. The statistical analysis of the rankings confirmed Simplatab as the top-performing framework. Simplatab significantly outperformed several frameworks, including H2O AutoML, PyCaret, TPOT, LightAutoML, and specific presets of MLjar (explain and compete) and Autogluon (medium). Conversely, frameworks such as Autogluon (good [*P*=.37], high [*P*=.60], and best [*P*=.56] presets) and MLjar (perform [*P*=.70] and optuna [*P*=.51]) showed no statistically significant difference in overall ranking compared with the top-performing framework, although they yielded lower absolute mean AUCs and generally required longer execution times.

Analysis of the median paired differences (Δ vs Ref) revealed the clinical and technical performance gaps between methods. For instance, the gap between Simplatab and LightAutoML, which showed promise in stable datasets (Table S4 in Multimedia Appendix 1), widened when evaluated across the full heterogeneous suite, showing a median deficit of 3.9% (95% CI 2.7%-8.6%). H2O AutoML and PyCaret exhibited the most severe underperformance, with median gaps of 6.7% and 7.8%, respectively.

In line with the exploratory suitability analysis (Figure 3), CRLM and Melanoma yielded poor performance across most frameworks (AUC≤60% with high SD), reflecting the intrinsic instability of these datasets. Nevertheless, Simplatab showed relatively greater robustness on these challenging scenarios, achieving modestly higher AUC values (around 64.5% and 65.6%, respectively) with lower variance, which suggests improved tolerance to noisy and unstable data. In contrast, framework performance was relatively homogeneous in low-differentiation datasets, confirming their limited utility for methodological discrimination. For instance, AUC differences across frameworks in the Lung and TCIA datasets were minimal, further corroborating the findings derived from Figure 3.

**Figure 4:**
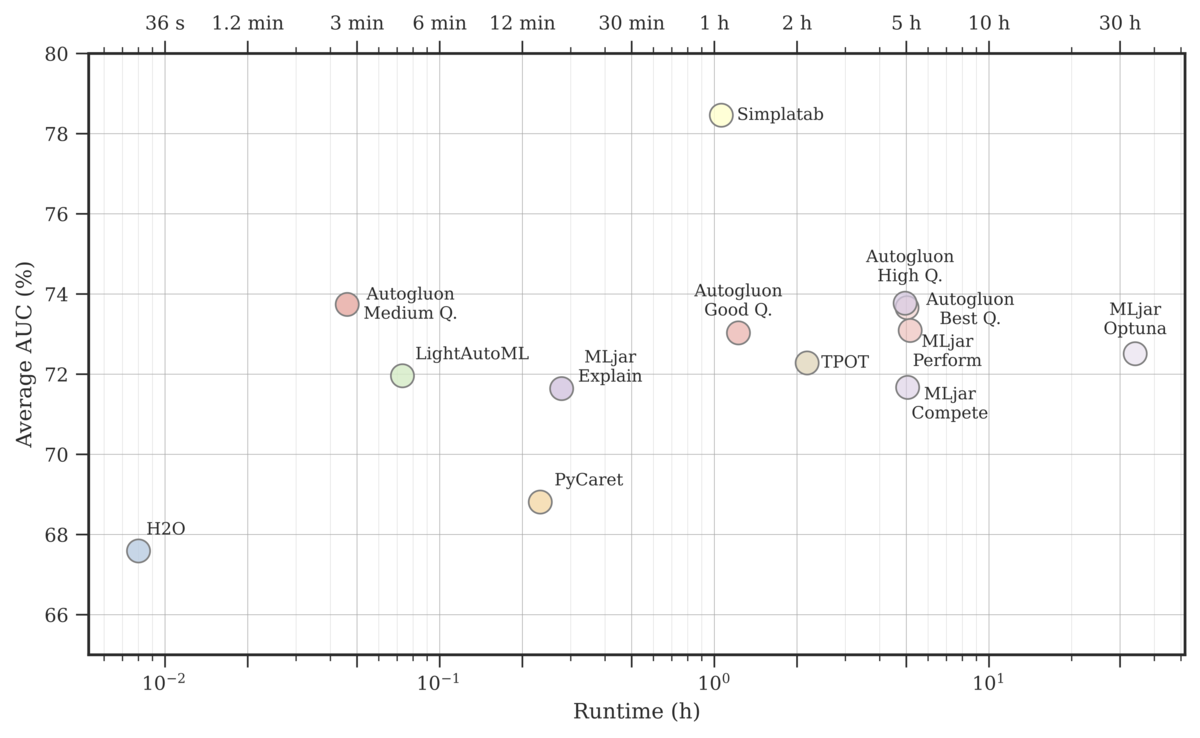
Performance and computational efficiency trade-offs of the evaluated automated machine learning frameworks. Test average area under the receiver operating characteristic curve (AUC; %) across all datasets is plotted against total runtime (h) on a logarithmic scale. The upper axis displays representative runtime values in natural scale to aid interpretation. Different presets of Autogluon and MLjar are shown separately. TPOT: Tree-Based Pipeline Optimization Tool.

Figure 4 presents the relationship between average AUC and runtime for each AutoML framework. From this joint perspective, a clear efficiency frontier emerges that is dominated by Simplatab. It occupies the most optimal position, providing the highest predictive performance with a moderate runtime (1.1 h). Fast alternatives, such as Autogluon medium, LightAutoML, and H2O, achieved speed at the cost of significant predictive accuracy. Meanwhile, the frameworks that achieved statistical parity with Simplatab in the ranking analysis (eg, Autogluon Best and MLjar optuna) fall behind on the efficiency frontier, requiring from 1.5 to 34 hours to complete the training process without surpassing Simplatab’s performance.

## Discussion

The comparative analysis of general-purpose and radiomics-specific AutoML frameworks for radiomics-based classification tasks reveals significant heterogeneity in performance, efficiency, computational costs, accessibility, and technical maturity, providing practical insights into the current capabilities of AutoML for radiomics applications.

A primary finding was Simplatab’s strong performance, achieving the highest average AUC across datasets (mean 78.46%, SD 12.22%), although differences in predictive performance were not consistently statistically significant compared with several general-purpose frameworks. Furthermore, it showed computational efficiency (1.1 h runtime) and relative resilience on challenging datasets such as CRLM and Melanoma, which exhibited high intramethod SD and poor performance across most other frameworks. Beyond performance, a notable characteristic of Simplatab is its accessibility and suite of tools, aimed at facilitating model inspection. The framework provides a graphical user interface that requires no coding expertise, together with built-in modules for model interpretability and basic assessments of data imbalance and model behavior. While these tools do not provide causal interpretability, they may represent practical mechanisms to increase model transparency and support user trust during model exploration and validation that may facilitate the gradual integration of radiomics-based models into clinical research workflows.

In contrast, general-purpose AutoML frameworks showed varying strengths and weaknesses. Intensive presets such as Autogluon (best and high quality) and MLjar (optuna) achieved statistical parity with Simplatab in overall dataset rankings; however, this competitive predictive performance came at the cost of substantially longer runtimes, which may limit their practicality for fast iteration. Autogluon medium quality, while emerging as an exceptionally fast option, exhibited a significant median performance gap (4.6%) compared with the top performer across the fully heterogeneous datasets. This positions Autogluon medium as a suitable tool for fast prototyping and preliminary analyses, but potentially suboptimal for final model selection. Conversely, H2O, despite being very fast, yielded lower predictive performance under default configurations, suggesting that its standard pipelines may be less suited for the complexities often inherent in high-dimensional radiomics datasets.

Our qualitative assessment highlighted significant limitations among several radiomics-specific tools. Frameworks such as AutoRadiomics and AutoML for Radiomics appeared to be inactive or technologically obsolete, reflecting the challenge of maintaining specialized software in a rapidly evolving machine learning landscape. WORC, while actively maintained, relied on configuration files and scripting that demanded considerable programming expertise, which conflicts with the low-code or no-code paradigm typically associated with AutoML solutions. AutoPrognosis, despite its advanced methodological scope, proved computationally infeasible for the high-dimensional datasets considered in this study under the adopted experimental constraints. Consequently, Simplatab was the only radiomics-specific framework that could be included under the constraints of this study.

The dataset suitability analysis revealed limitations in several public radiomics datasets: CRLM and Melanoma showed high intramethod variability, making it difficult to discern true performance differences from statistical noise, whereas others (Lung, TCIA, and Liver) showed low intermethod variability, which limits their ability to discriminate between modeling pipelines. These findings suggest the need for larger, more diverse, and statistically stable radiomics datasets to support meaningful methodological comparisons and advancements.

Our study has limitations that highlight critical gaps in the current AutoML landscape for radiomics. First, the present evaluation focused exclusively on classification tasks. The absence of robust, user-friendly AutoML frameworks supporting survival analysis represents a major bottleneck for oncology, where time-to-event end points are paramount. Future development should prioritize the integration of survival models into accessible AutoML packages. In addition, because the primary objective of this study was methodological comparison across heterogeneous datasets rather than specific clinical deployment, our evaluation relied primarily on threshold-independent discrimination (AUC). Assessing true clinical translation requires calibration metrics and decision-curve analysis, which depend on disease-specific contexts and operational thresholds. The absence of these context-dependent metrics remains a limitation, and future studies must evaluate calibration and net clinical benefit. Second, the use of pre-extracted features isolated the modeling component of the radiomics pipeline but does not address the ultimate goal of achieving a fully automated, end-to-end workflow. As a consequence, the exclusion of some tools due to compatibility or usability constraints may introduce a degree of selection bias. Additionally, minor framework-specific preprocessing adaptations (eg, imputation or encoding) were strictly required to prevent the exclusion of patients. While these adaptations were necessary to ensure that all frameworks were evaluated on the same cohorts, we acknowledge that such preprocessing differences may have influenced performance to some extent; however, their impact was not formally evaluated and therefore represents a limitation for the comparative study. A major unmet need is the development of frameworks that also integrate upstream steps such as feature extraction, harmonization, and reproducibility control, which remain critical barriers for clinical translation and are not within the scope of this study. While tools like Simplatab demonstrate promise at the modeling stage, to ensure reliability and clinical trust, the field should progress toward holistic solutions that automate the entire radiomics workflow, from image to prediction. Finally, it is important to highlight that these findings should be interpreted within the context of an internal benchmarking study, as no external validation was performed.

In conclusion, AutoML frameworks offer substantial potential to accelerate and democratize radiomics research by automating complex modeling tasks. While no single framework demonstrated absolute predictive superiority, Simplatab emerges as a promising tool for users prioritizing ease of use, model inspection, and strong predictive performance with reasonable computational cost. General-purpose tools such as the Autogluon medium preset provide efficient options for rapid experimentation, while heavier presets from Autogluon or MLjar achieved statistically comparable performance to Simplatab, but they required substantially higher computational times, highlighting the importance of the efficiency trade-offs. However, substantial challenges remain. The field requires more stable and diverse radiomics datasets, AutoML solutions capable of supporting survival analysis and, critically, frameworks that enable robust harmonization and reproducibility across the full radiomics workflow are required. Although these tools represent a step forward for the modeling stage, considerable development is still required to achieve fully automated, reliable, and clinically translatable radiomics pipelines.

## Data Availability

Open-source datasets from Open Radiomics and the Workflow for Optimal Radiomics Classification are publicly available in their respective repositories [30,31]. Private datasets (Lung and Prostate) are not publicly available due to data-sharing restrictions, as they contain information from multiple institutions and are not solely owned by our research group, but are available from the corresponding author on reasonable request. All code is available on GitHub [28].

## Appendix

Multimedia Appendix 1 Supplementary methodological details, dataset characteristics, CLAIM checklist, AutoML framework specifications, and complementary performance metrics for the comparative evaluation of automated machine learning frameworks in radiomics.

## Abbreviations

AUC: area under the receiver operating characteristic curve
AutoML: automated machine learning
BraTS: Brain Tumor Segmentation
CRLM: colorectal liver metastases
GIST: gastrointestinal stromal tumors
MR: magnetic resonance
TCIA: The Cancer Imaging Archive
TPOT: Tree-Based Pipeline Optimization Tool
WORC: Workflow for Optimal Radiomics Classification
