# Supplementary material for: Automated Machine Learning Frameworks for Radiomics: Comparative Evaluation Study
Source: JMIR Form Res. 2026 Jun 11;10:e91492. doi: 10.2196/91492 (PMC13305470; doi:10.2196/91492)
Supplement: Multimedia Appendix 1 [file formative_v10i1e91492_app1.docx]

Automated Machine Learning in Radiomics: A Comparative Evaluation of Performance, Efficiency and Accessibility

# Supplementary Material

## Radiomics Feature Extraction

Radiomic feature extraction was performed differently depending on the dataset's origin. For the publicly available datasets obtained from OpenRadiomics (*BraTS* and *TCIA*), pre-computed radiomics feature sets were utilized. Both feature sets were originally generated using a *binwidth* parameter of 25 with z-score normalization.

For the remaining evaluated datasets (C*RLM, Desmoid, GIST, Lipo, Liver, Melanoma, Lung, Prostate*), radiomics features were extracted using the PyRadiomics python library (v3.1.0) [1]. A standardized configuration pipeline was implemented in PyRadiomics, consisting of image resampling to isotropic voxel, z-score normalization to standardize intensity values, and removal of outliers deviating by more than three standard deviations from the mean within the region of interest to mitigate their potential influence.

Feature extraction included shape, first- and second-order features from the original image, along and high-order features derived from filtered image representations. The applied filters included: square, exponential, logarithm, wavelet transforms, and Laplacian of gaussian with sigma values specified as 0.5, 3.0, and 5.0. This extraction protocol yielded a total of 1379 distinct radiomic features per case.

## Experimental Setup

Experiments were executed on a computing environment comprising an Intel Xeon CPU E3-1240 v6 (4 cores at 3.70 GHz), 33 GB of RAM, running CentOS Linux 7. Python versions 3.8 or 3.10 were used, depending on compatibility requirements with the specific frameworks. The random seed was fixed across methods (7).

The reliance on a CPU-based environment for all experiments was intentional and reflects the nature of state-of-the-art AutoML for tabular data. The leading algorithms for tabular data, such as the gradient-boosted trees and ensembles that power most frameworks, are highly optimized for efficient CPU execution. While GPU support exists for some of these algorithms, the performance benefits are typically only realized on datasets far larger than those common in radiomics.

## Datasets

### Public datasets sources

- OpenRadiomics datasets:
  - BraTS: <https://openradiomics.org/?page_id=1163>
  - TCIA: <https://openradiomics.org/?page_id=1144>
- WORC datasets: <https://xnat.health-ri.nl/data/projects/worc>

### Variable response distribution

**Table S1.** Summary of response variable distributions for each dataset.

| Dataset | Response Variable Description | Distribution [n] | |
| --- | --- | --- | --- |
| BraTS | MGMT | Non-methylated = 276  Methylated = 301 | |
| TCIA | TNM Overall Stage | I = 93  II = 40  IIIa = 112  IIIb = 176 | *Binarized:*  Stage I + II = 133  Stage III = 288 |
| CRLM | Histological Growth Pattern (HGP) | Desmoplastic HGP = 38  Replacement HGP = 36 | |
| Desmoid | Tumor Type | Soft Tissue Sarcoma (STS) = 131  Desmoid-Type Fibromatosis (DTF) = 71 | |
| GIST | Tumor Type | Non-GIST = 121  Gastrointestinal Stromal Tumor (GIST) = 125 | |
| Lipo | Tumor Type | Lipoma = 57  Well-Differentiated Liposarcoma (WDLPS) = 57 | |
| Liver | Lesion Malignancy | Malignant = 91  Bening = 94 | |
| Melanoma | BRAF Mutation Status | BRAF Mutated = 50  BRAF Wild Type = 52 | |
| Lung | Survival Status at 12 Months | Survived = 260  Deceased = 294 | |
| Prostate | Gleason Score | 6 = 78  7 = 200  8 = 24  9 = 28  10 = 2 | *Binarized:*  < 7 = 78  ≥ 7 = 252 |
| Abbreviations: MGMT, O^6^-methylguanine-DNA methyltransferase; TNM, Tumor - Node - Metastasis staging; BRAF, v-raf murine sarcoma viral oncogene homolog B1 gene. | | | |

**CLAIM: Checklist for Artificial Intelligence in Medical Imaging**

**Table S2.** Checklist for Artificial Intelligence in Medical Imaging (CLAIM) 2024 Update [2]. X: item included in the study; No: item not included in the study; NA: not applicable to this study.

| **Section / Topic** | **No.** | **Item** |  |
| --- | --- | --- | --- |
| TITLE / ABSTRACT |  | | |
|  | **1** | Identification as a study of AI methodology, specifying the category of technology used (e.g., deep learning) | **X** |
|  | **2** | Structured summary of study design, methods, results, and conclusions | **X** |
| INTRODUCTION |  |  |  |
|  | **3** | Scientific and clinical background, including the intended use and clinical role of the AI approach | **X** |
|  | **4** | Study objectives and hypotheses | **X** |
| METHODS |  |  |  |
| *Study Design* | **5** | Prospective or retrospective study | **NA** |
|  | **6** | Study goal | **X** |
| *Data* | **7** | Data sources | **X** |
|  | **8** | Inclusion and exclusion criteria | **X** |
|  | **9** | Data pre-processing steps | **X** |
|  | **10** | Selection of data subsets | **NA** |
|  | **11** | De-identification methods | **NA** |
|  | **12** | How missing data were handled | **X** |
|  | **13** | Image acquisition protocol | **X** |
| *Reference Standard* | **14** | Definition of method(s) used to obtain reference standard | **X** |
|  | **15** | Rationale for choosing the reference standard | **NA** |
|  | **16** | Source of reference standard annotations | **NA** |
|  | **17** | Annotation of test set | **NA** |
|  | **18** | Measures of inter- and intra-rater variability of features described by the annotators | **NA** |
| *Data Partitions* | **19** | How data were assigned to partitions | **X** |
|  | **20** | Level at which partitions are disjoint | **X** |
| *Testing Data* | **21** | Intended sample size | **X** |
| *Model* | **22** | Detailed description of model | **X** |
|  | **23** | Software libraries, frameworks, and packages | **X** |
|  | **24** | Initialization of model parameters | **X** |
| *Training* | **25** | Details of training approach | **X** |
|  | **26** | Method of selecting the final model | **X** |
|  | **27** | Ensembling techniques | **NA** |
| *Evaluation* | **28** | Metrics of model performance | **X** |
|  | **29** | Statistical measures of significance and uncertainty | **X** |
|  | **30** | Robustness or sensitivity analysis | **X** |
|  | **31** | Methods for explainability or interpretability | **X** |
|  | **32** | Evaluation on internal data | **X** |
|  | **33** | Testing on external data | **No** |
|  | **34** | Clinical trial registration | **NA** |
| RESULTS |  |  |  |
| *Data* | **35** | Numbers of patients or examinations included and excluded | **NA** |
|  | **36** | Demographic and clinical characteristics of cases in each partition | **NA** |
| *Model performance* | **37** | Performance metrics and measures of statistical uncertainty | **X** |
|  | **38** | Estimates of diagnostic accuracy and their precision | **X** |
|  | **39** | Failure analysis of incorrectly classified cases | **No** |
| DISCUSSION |  |  |  |
|  | **40** | Study limitations | **X** |
|  | **41** | Implications for practice, including the intended use and/or clinical role | **X** |
| OTHER INFORMATION |  |  |  |
|  | **42** | Provide a reference to the full study protocol or to additional technical details | **X** |
|  | **43** | Statement about the availability of software, trained model, and/or data | **X** |
|  | **44** | Sources of funding and other support; role of funders | **X** |
| X = item included in the study; No = item not included in the study; NA = not applicable | | | |

## AutoML Frameworks Pipelines

**Table S3a**. Comparative summary of the AutoML frameworks evaluated in the performance comparison based on feature selection strategies, supported machine learning models, and additional functionalities

| Framework | Feature Selection | Machine Learning Models | Additional Functionalities |
| --- | --- | --- | --- |
| Autogluon  (v.1.1.0) | No advanced feature selection by default; removes constant and duplicate features. | LightGBM, XGBoost, CatBoost, Random Forest, KNN, logistic regression, PyTorch and FastAI neural networks; uses ensemble methods (stacking). | Preset configurations balance accuracy and speed with hyperparameter search**:**  *- Medium:* default preset for fastest training/inference and lowest accuracy.  *- Good:* fast with good accuracy (~4× faster than *High*).  *- High:* strong accuracy with moderate speed.  *- Best:* maximum accuracy, slowest training/inference. |
| MLjar  (v.1.1.9) | *-* *Explain:* No feature selection.  *- Others preset:* Automatic iterative feature selection using permutation-based importance with random feature comparison across multiple learners. | Linear/logistic regression, decision trees, Random Forest, ExtraTrees, LightGBM, XGBoost, CatBoost, neural networks, KNN; combines top performers using ensemble methods and hill-climbing optimization. | - *Explain:* maximum interpretability (learning curves, importance plots, and SHAP plots) with minimal computation (minimal tuning with only 1 model per algorithm using default hyperparameters). Fastest option with basic 75%/25% train/test split.  - Perform: moderate explanations (learning curves and importance plots only) with balanced tuning (13 total models, including some hyperparameter optimization). 5-fold cross-validation for better performance estimation  *- Compete:* No explanations generated with extensive tuning (22 total models and aggressive hyperparameter search) 10-fold cross-validation for most robust evaluation with advanced ensemble methods (stacking and ensemble stacked).  *- Optuna*: c*ompete* preset with intensive tuning using Optuna. |
| Abbreviations: CV, Cross-Validation; DNN, Deep Neural Network; GLM, Generalized Linear Model; KNN, K-Nearest Neighbors, MLP, Multilayer Perceptron; RFE, Recursive Feature Elimination; SGD; Stochastic Gradient Descent; SHAP, Shapley Additive exPlanations; SVM, Support Vector Machine, XRT, eXtra Random Trees. | | | |

**Table S3b.** Comparative summary of the AutoML frameworks evaluated in the performance comparison based on feature selection strategies, supported machine learning models, and additional functionalities.

| Framework | Feature Selection | Machine Learning Models | Additional Functionalities | |
| --- | --- | --- | --- | --- |
| H2O  (v.3.46.0) | No additional feature selection; performs automatic target encoding for high-cardinality categorical variables. | Multiple H2O-native algorithms: GLM with regularization, XGBoost, LightGBM, Random Forest, DNN, XRT; produces stacked ensembles. | Parallel model execution with early stopping; produces ensembles with regularized meta-learner. | |
| LightAutoML  (v.0.4.1) | Feature preselection based on importance and sequential selection | Multi-level pipelines with LightGBM, CatBoost, Random Forest; uses stacking with weighted blending. | Automatic hyperparameter tuning with Optuna and cross-validation. | |
| PyCaret  (v.3.3.2) | Feature selection through SelectFromModel (default: LightGBM), SelectKBest, or SequentialFeatureSelector; includes multicollinearity removal. | Wide range of scikit-learn and boosting algorithms: linear/logistic regression, KNN, SVM, Naive Bayes, decision trees, Random Forest, ExtraTrees, AdaBoost, Gradient Boosting, LightGBM, CatBoost, XGBoost, MLP; supports basic ensembling. | Hyperparameter tuning with random search and cross-validation. | |
| Simplatab  (v.1.0.0) | Removes highly correlated features, then applies combined approach: SULOV (stability selection) + RFE using XGBoost as base model. | Seven classifiers: logistic regression, decision trees, Random Forest, XGBoost, multilayer perceptron (MLP), SGD classifier, SVM; hyperparameters optimized with cross-validation. | Includes bias detection and model vulnerability analysis. | |
| TPOT  (v.1.1.0) | Genetic feature selection during evolutionary pipeline search, incorporating filtering nodes. | Scikit-learn estimators: trees, SVM, KNN, linear regression, simple neural networks. | Uses Genetic Programming to evolve pipelines with crossover/mutation for objective metric improvement. | |
| Abbreviations: CV, Cross-Validation; DNN, Deep Neural Network; GLM, Generalized Linear Model; KNN, K-Nearest Neighbors, MLP, Multilayer Perceptron; RFE, Recursive Feature Elimination; SGD; Stochastic Gradient Descent; SHAP, Shapley Additive exPlanations; SVM, Support Vector Machine, XRT, eXtra Random Trees. | | | |  |

## Complementary Metrics

**Table S4.** Additional metrics of the comparative evaluation of AutoML frameworks. Average performance across datasets from 5-fold nested cross validation (mean ± standard deviation). Below each average, the median paired difference (Δ Ref) for each metric compared to the top-performing framework (Simplatab) is provided, along with its 95% confidence interval (CI). Statistical comparisons and effect size measures are indicated.

| Average (%)  Δ Ref. [95% CI] | AUC | F1 | Balanced Accuracy | Specificity | Sensitivity |
| --- | --- | --- | --- | --- | --- |
| Autogluon – Medium | 73.72 ± 12.81 ^*^  4.6 [3.0, 6.5] | 66.15 ± 15.6  -1.0 [-8.0, 8.2] | 66.8 ± 11.13  4.1 [-12.2, 16.2] | 69.35 ± 23.84  -4.5 [-25.7, 19.8] | 64.24 ± 19.01  6.7 [-13.7, 22.3] |
| Autogluon – Good | 73.02 ± 15.31  3.8 [2.1, 8.7] | 66.57 ± 16.6  -2.0 [-10.3, 7.4] | 67.12 ± 12.16  2.0 [-13.5, 18.2] | 68.78 ± 20.64  -3.4 [-23.7, 20.8] | 65.45 ± 19.37  2.7 [-16.9, 24.3] |
| Autogluon – High | 73.08 ± 16.02  2.8 [2.4, 9.1] | 65.38 ± 18.72  -1.5 [-11.7, 12.1] | 66.59 ± 14.43  -0.3 [-13.6, 19.8] | 68.77 ± 20.29  1.1 [-25.9, 21.7] | 64.41 ± 18.68  5.4 [-17.1, 23.2] |
| Autogluon – Best | 73.66 ± 14.77  3.5 [1.9, 7.2] | 65.35 ± 19.37  -3.0 [-10.5, 9.5] | 66.73 ± 12.94  2.4 [-14.6, 18.0] | 67.85 ± 20.98  0.6 [-24.4, 19.7] | 65.61 ± 20.61  2.4 [-19.2, 23.1] |
| MLjar –  Explain | 71.64 ± 13.98 ^*^  6.2 [4.4, 8.3] | 65.24 ± 15.56  2.2 [-5.4, 8.9] | 64.95 ± 11.67  5.5 [-12.6, 18.9] | 62.40 ± 21.98  5.6 [-17.4, 26.9] | 67.51 ± 18.45  1.0 [-18.5, 17.7] |
| MLjar – Perform | 73.78 ± 13.99  3.8 [2.5, 6.1] | 68.16 ± 14.88  -3.3 [-9.5, 7.3] | 66.22 ± 12.96  5.0 [-15.1, 18.1] | 61.84 ± 23.26  9.9 [-17.6, 25.2] | 70.59 ± 17.24  -6.0 [-20.9, 20.2] |
| MLjar – Compete | 71.67 ± 13.14 ^*^  6.4 [3.8, 9.0] | 66.4 ± 13.44  -3.5 [-6.5, 7.9] | 66.01 ± 10.07  1.4 [-13.7, 18.0] | 62.26 ± 15.01  5.1 [-10.2, 19.2] | 69.76 ± 13.9  -1.4 [-21.7, 18.1] |
| MLjar –  Optuna | 72.51 ± 16.19 ^*^  3.7 [2.3, 7.9] | 65.7 ± 18.45  -2.5 [-10.1, 10.1] | 65.4 ± 14.25  4.0 [-14.1, 18.8] | 63.69 ± 22.57  7.3 [-19.6, 23.6] | 67.12 ± 21.44  -1.5 [-22.5, 26.3] |
| H2O AutoML | 67.59 ± 15.10 ^*^  6.7 [5.0, 14.5] | 69.2 ± 10.27  -4.3 [-7.8, 0.4] | 63.42 ± 12.24  9.2 [-10.5, 18.1] | 45.95 ± 34.83  18.7 [-11.6, 63.2] | 80.89 ± 17.05  -20.7 [-27.7, 10.4] |
| LightAutoML | 71.95 ± 17.29 ^*^  3.9 [2.7, 8.6] | 62.17 ± 20.95  0.0 [-8.4, 14.9] | 64.45 ± 13.32  8.2 [-10.2, 16.9] | 68.42 ± 26.97  -6.0 [-25.2, 19.9] | 60.47 ± 24.78  9.6 [-16.7, 36.9] |
| PyCaret | 68.80 ± 16.83 ^*^  7.8 [4.8, 13.7] | 64.31 ± 16.78  0.2 [-8.7, 10.1] | 63.91 ± 13.42  6.3 [-9.8, 19.5] | 63.82 ± 25.27  4.2 [-19.1, 22.7] | 64.01 ± 18.94  6.7 [-15.2, 23.7] |
| Simplatab  *(Reference)* | 78.46 ± 12.22 | 65.75 ± 10.68 | 68.02 ± 10.20 | 67.96 ± 10.35 | 68.09 ± 10.14 |
| TPOT | 72.28 ± 14.46 ^*^  5.4 [3.9, 7.7] | 63.8 ± 18.48  -1.3 [-8.4, 13.5] | 65.08 ± 12.97  4.5 [-10.6, 19.7] | 68.36 ± 22.2  0.3 [-22.2, 17.2] | 61.79 ± 21.15  6.2 [-14.5, 33.0] |
| *Friedman Test (P)*  *Kendall’s W* | 5.7⋅10^-7^  0.568 (strong) | 0.626  0.108 (small) | 0.213  0.169 (small) | 0.002  0.332 (moderate) | 0.134  0.189 (small) |
| ^*^ Statistically significant difference compared to Simplatab from Nemenyi post-hoc test *(P<.05)* | | | | | |

**Table S5.** Additional metrics of the comparative evaluation of AutoML frameworks, excluding *CRLM* and *Melanoma* datasets, due to the lack of reliable performance across frameworks. Average performance across datasets from 5-fold nested cross validation (mean ± standard deviation). Below each average, the median paired difference (Δ Ref) for each metric compared to the top-performing framework (Simplatab) is provided, along with its 95% confidence interval (CI). Statistical comparisons and effect size measures are indicated.

| Average (%)  Δ Ref. [95% CI] | AUC | F1 | Balanced Accuracy | Specificity | Sensitivity |
| --- | --- | --- | --- | --- | --- |
| Autogluon – Medium | 77.19 ± 11.78 ^*^  4.6 [3.0, 6.5] | 69.39 ± 15.87  -2.4 [-13.2, 8.2] | 69.35 ± 10.96  -1.6 [-11.1, 17.1] | 71.37 ± 26.12  -7.2 [-19.0, 19.9] | 67.32 ± 20.14  -0.6 [-15.5, 12.9] |
| Autogluon – Good | 78.25 ± 12.03  3.2 [1.8, 5.9] | 71.79 ± 13.95  -5.5 [-14.3, 1.3] | 70.81 ± 10.57  -3.6 [-12.2, 15.1] | 70.32 ± 22.67  -4.3 [-14.4, 20.3] | 71.30 ± 16.63  -7.0 [-17.5, 9.8] |
| Autogluon – High | 78.74 ± 12.14  2.6 [2.3, 4.4] | 71.79 ± 14.14  -4.7 [-15.8, 1.6] | 71.57 ± 11.2  -2.7 [-14.7, 17.8] | 73.26 ± 20.31  -6.2 [-18.9, 18.1] | 69.87 ± 16.42  -4.7 [-14.6, 17.5] |
| Autogluon – Best | 78.37 ± 12.32  2.9 [1.5, 5.6] | 71.54 ± 15.6  -6.5 [-13.6, 5.8] | 70.73 ± 11.13  -4.1 [-13.1, 17.3] | 70.34 ± 22.85  -6.0 [-15.1, 22.5] | 71.13 ± 18.99  -8.8 [-17.6, 12.2] |
| MLjar –  Explain | 76.09 ± 11.67 ^*^  4.9 [4.1, 7.8] | 68.57 ± 15.72  -1.9 [-6.5, 8.9] | 67.63 ± 11.52  -1.0 [-10.4, 18.2] | 64.58 ± 24.37  2.6 [-11.9, 27.5] | 70.68 ± 19.33  -8.0 [-16.5, 8.9] |
| MLjar – Perform | 78.30 ± 11.50  3.7 [2.2, 4.6] | 72.57 ± 13.12  -6.4 [-11.3, 1.7]] | 69.65 ± 12.18  -5.4 [-13.3, 20.3] | 63.67 ± 26.0  0.9 [-8.3, 31.5] | 75.63 ± 15.34  -10.2 [-19.3, 4.9] |
| MLjar – Compete | 76.14 ± 10.33 ^*^  5.2 [3.6, 7.2] | 70.48 ± 11.58  -4.6 [-7.9, 1.5] | 68.99 ± 8.92  -2.9 [-10.2, 16.8] | 63.78 ± 16.58  0.6 [-8.7, 21.7] | 74.20 ± 11.60  -9.9 [-16.1, 15.8] |
| MLjar –  Optuna | 78.61 ± 10.85  3.2 [1.8, 4.3] | 71.77 ± 14.91  -5.9 [-13.4, 4.4] | 70.41 ± 10.82  -3.6 [-11.5, 16.7] | 67.14 ± 24.21  -2.4 [-13.7, 23.2] | 73.68 ± 18.36  -9.8 [-15.8, 3.9] |
| H2O AutoML | 71.99 ± 13.52 ^*^  6.1 [4.2, 9.2] | 70.1 ± 11.45  -4.5 [-10.9, 4.1] | 66.77 ± 11.33  5.7 [-12.2, 15.9] | 57.44 ± 28.38  8.4 [-11.1, 39.7] | 76.11 ± 15.6  -8.7 [-18.7, 11.5] |
| LightAutoML | 78.19 ± 12.43  3.1 [2.4, 5.1] | 67.6 ± 19.54  -3.9 [-9.4, 9.2] | 67.96 ± 11.93  1.8 [-14.6, 21.5] | 69.24 ± 29.95  -9.4 [-15.9, 24.3] | 66.68 ± 23.78  -5.1 [-15.8, 19.7] |
| PyCaret | 74.12 ± 14.0 ^*^  6.6 [3.6, 8.5] | 68.91 ± 15.25  -2.8 [-12.0, 6.0] | 68.03 ± 11.57  1.0 [-11.6, 20.8] | 67.71 ± 27.02  -3.5 [-13.4, 20.3] | 68.35 ± 18.39  -3.7 [-13.5, 16.1] |
| Simplatab  *(Reference)* | 81.81 ± 11.30 | 66.75 ± 11.86 | 71.62 ± 9.12 | 71.48 ± 8.93 | 71.76 ± 9.35 |
| TPOT | 76.88 ± 11.89 ^*^  4.5 [3.6, 6.3] | 69.59 ± 15.67  -3.5 [-12.4, 7.7] | 69.28 ± 10.72  -1.4 [-13.2, 16.3] | 70.67 ± 24.54  -6.9 [-15.2, 20.0] | 67.88 ± 19.06  -3.0 [-13.3, 12.5] |
| *Friedman Test (P)*  *Kendall’s W* | 2.0⋅10^-5^  0.835 (strong) | 0.061  0.347 (moderate) | 0.061  0.348 (moderate) | 0.078  0.310 (moderate) | 0.080  0.330 (moderate) |
| ^*^ Statistically significant difference compared to Simplatab from Nemenyi post-hoc test *(P<.05)* | | | | | |

# References

[1] J. J. M. van Griethuysen *et al.*, “Computational Radiomics System to Decode the Radiographic Phenotype,” *Cancer Research*, vol. 77, no. 21, pp. e104–e107, Nov. 2017, doi: 10.1158/0008-5472.CAN-17-0339.

[2] A. S. Tejani, M. E. Klontzas, A. A. Gatti, J. T. Mongan, et al. “Checklist for Artificial Intelligence in Medical Imaging (CLAIM): 2024 Update,” *Radiology: Artificial Intelligence*, Accessed: May 11, 2026. [Online]. Available: https://pubs.rsna.org/doi/10.1148/ryai.240300.
